# Pilot Study: Simultaneous Daily Recording of Total Locomotor Activity and Heart Rate in Horses for Application in Precision Livestock Farming

**DOI:** 10.3390/ani15091189

**Published:** 2025-04-22

**Authors:** Francesca Aragona, Maria Rizzo, Federica Arrigo, Francesca Arfuso, Francesco Fazio, Elisabetta Giudice, Pietro Pugliatti, Giuseppe Piccione, Claudia Giannetto

**Affiliations:** 1 Department of Veterinary Sciences, University of Messina, Viale Giovanni Palatucci snc, 98168 Messina, Italy; francesca.aragona@unime.it (F.A.); federica.arrigo@studenti.unime.it (F.A.); farfuso@unime.it (F.A.); ffazio@unime.it (F.F.); egiudice@unime.it (E.G.); gpiccione@unime.it (G.P.); claudia.giannetto1@unime.it (C.G.); 2 Department of Clinical and Experimental Medicine, University of Messina, 98168 Messina, Italy; pietro.pugliatti@unime.it

**Keywords:** locomotor activity, heart rate, equine, precision livestock farming, welfare

## Abstract

Among physiological parameters, total locomotor activity (TLA) and heart rate (HR) are used as physiological indicators in animal welfare evaluations. The present study aimed to simultaneously record for 24 h the TLA and HR of ten clinically healthy horses housed in conventional individual boxes subjected to a natural photoperiod and temperature. An actigraphy-based data logger was placed on the headstall, and an equine HR monitor was placed around the chest to monitor TLA and HR, respectively. Activity was monitored with 5 min sampling intervals and HR with 5 s intervals. To make the data points uniform, the means of 5 min intervals were calculated. Both investigated parameters showed a daily rhythmicity with a diurnal acrophase (locomotor activity 17:05 ± 1:15 arbitrary unit; heart rate 16.40 ± 0.30 beats/min). Robustness of the rhythm was 17.95 ± 10.53% and 37.05 ± 0.63% for the TLA and HR. A positive correlation was observed between the two investigated parameters in each horse, r = 0.48 ± 0.07, *p* < 0.0001. Change in TLA is a good index for success of management. Its positive correlation with daily HR monitoring confirms the use of these two physiological parameters for an objective on-farm welfare assessment. The application of new technologies for the simultaneous recording of physiological indexes of animals’ welfare can be a useful instrument.

## Simple Summary

Total locomotor activity and heart rate are important physiological parameters for assessing animal welfare. In this study, ten horses were enrolled, and these parameters were recorded simultaneously for 24 h. An actigraphy-based data recorder was placed on the head, and an equine heart rate monitor was placed around the chest. Both investigated parameters showed a daily rhythmicity with a diurnal acrophase (locomotor activity 17:05 ± 1:15 arbitrary unit; heart rate 16.40 ± 0.30 beats/min). The rhythm robustness was 17.95 ± 10.53% and 37.05 ± 0.63% for locomotor activity and heart rate, respectively. A positive correlation was found in both parameters. This indicates that the use of the new technology is a useful instrument for the assessment of animal welfare.

## 1. Introduction

Animal welfare is the state of an animal’s body and the mind and level to which its requirements are satisfied [1], and it can be measured by means of behavioural, postural and physiological parameters [2]. Precision livestock farming (PLF) is one of the most powerful developments amongst several interesting new and upcoming technologies that have the potential to revolutionise the livestock farming industry.

Various PLF applications have been developed over the past two decades, including precision grazing technologies and management software support tools [3,4,5], image analysis methods for grazing measurements [6], electronic identification systems such as radio frequency identification (RFID) tags [7,8], movement detection systems including accelerometers [9,10] and GPS [11], audio analysis systems [12], flock management systems such as virtual fencing [13] and drones [14] and health detection and welfare assessment systems with the use of implanted sensors [15].

One of the objectives of PLF is to improve or at least objectively document, with the help of numerical data, animal welfare on farms [16]. Even though principally applied to animals in production, the use of technology for welfare monitoring could be applied to other species, such as horses. Therefore, the first step in creating a PLF model is behavioural analysis through animal-based observations [17].

Physiological parameters, such as heart rate, blood pressure, body temperature, serum levels of various stress hormones (e.g., cortisol) and immunological functions (e.g., suppression of lymphocyte activity) can be used to assess welfare. The measurement of many of these parameters requires invasive monitoring techniques. However, where instrumentation, such as actigraphy-based data loggers and heart rate transmitters, has already been used for experimental purposes, data that can help to assess welfare can be obtained with no additional adverse effects for the animal [2]. In equines, the main behaviours carried out throughout the day include feeding, drinking, walking and grooming [18]. These behaviours, together with the small movements performed during the dark phase of the light/dark cycle, are defined as total locomotor activity (TLA) and are primarily influenced in domestic contexts by management systems such as housing and feeding practices [19]. Total locomotor activity has been monitored in various horse breeds as well as different environmental and housing conditions [20,21].

It is well known that TLA has a daily rhythmicity, which is different from species to species [18]. Horses show a circadian rhythm in their activity and are mainly diurnal animals as their TLA has been demonstrated to have its peak during the photophase [18]. Also, a seasonal variation of TLA has been demonstrated in sport horses, with an effect of photoperiod influencing the daily amount of activity that was highest during the warmer months [22]. However, throughout the year, the activity peak was consistently recorded in the middle of the photophase, with no recorded difference in the acrophase.

One of the most common approaches to evaluating the horses psychosomatic response to environmental effect is the assessment of HR [23]. Over the past decades, the advance in biologging technologies has allowed for the long-term collection of previously inaccessible physiological data, such as HR monitoring [24,25]. Changes in this physiological parameter reflect autonomic nervous system dynamics [26], revealing information on the response of an individual animal to a given situation. In horses, it has been demonstrated that heart rate has a diurnal daily rhythm with low robustness, indicating a low stability of the rhythm. Exposure of horses to novelty and just the expectation of novelty has been demonstrated to increase HR, such as moving horses in a new training centre or keeping stallion and mares in the same stable. On the contrary, Janczarek et al. [27] demonstrated that keeping racehorses in a paddock in an off-the-racetrack centre resulted in a better psychosomatic state of animals as assessed by HR monitoring in comparison with animals living in the racetrack centre. The relationship between spontaneous locomotor activity and cardiovascular endpoints has never been subjected to a careful analysis that would allow for the quantitative estimation of the contribution of locomotor activity to minute-by-minute heart rate with any degree of precision. Equine behaviour, as well as physiological status, are good indicators of welfare [28], but there is a need for objective and quantifiable assessment tools [28]. The scope of PLF is to improve animal welfare; most of the systems monitor just one or only a few factors. The results of these measurements are compared with a general standard or farm-specific threshold, and a conclusion or alert is communicated to the farmer, prompting them to check the animal and take action if necessary. A range of PLF sensors have been developed to improve the efficiency of animal production by optimising management. Data from these sensors could be integrated into automated welfare assessment systems. An integrated approach to animal welfare assessments should be possible, but this approach needs to be further defined and validated [29].

The accelerometer is a sensor capable of measuring variations in acceleration along one or multiple reference axes. It is a widely used tool in farming, providing valuable data and insights on animal activity levels, behaviour patterns and health status. Small devices equipped with accelerometers are attached to the animal, often integrated into collars or harnesses. The placement and parameter configuration of the accelerometer are determined according to specific research objectives. The impact of rest and sleep quality on the overall animal welfare remains an area that requires more in-depth investigation, considering the importance it has for maintaining homeostasis and development. Understanding how these aspects influence animal health and development is crucial for improving welfare standards and ensuring better management practices. Moreover, several works have evaluated the impact of management practices, feeding, housing, sickness and pain on lying behaviour. An increase in lying behaviour was found in both favourable conditions (comfort) and unfavourable conditions (sickness/pain), but also decreased in favourable conditions (like social housing—animals are more active and play); this implies that the use of this indicator should be carefully considered, and researchers should take account of multiple aspects when considering it. It is also necessary to establish a threshold on healthy animals, considering the evolution in the resting time budget [30]. Combining accelerometry with physiological data from HR loggers supports more comprehensive welfare monitoring, facilitating data-driven management in extensive systems. The implementation of HR loggers presents practical challenges: external factors—such as environmental conditions, stressors, individual temperament and overall health—can influence HR independently of movement, underscoring the importance of careful interpretation when using movement proxies for physiological data [31].

The aim of the present study was to simultaneous record TLA and HR to verify their possible correlation in horses housed in conventional boxes by means of an actigraphy-based data logger and an HR monitor for a possible application in PLF in this species.

## 2. Materials and Methods

Our study used ten clinically healthy Italian saddle horses (gelding, 8–10 years old, mean body weight 415 ± 25 Kg) in good nutritional condition (the Henneke Body Condition Scoring System = 5). Before the start of the study, all animals underwent a clinical exam to establish their health status. Their health status was evaluated based on heart rate, respiratory rate, appetite, faecal consistency, and haematologic and haematochemical profile. Animals were housed individually in their usual 12 m^2^ boxes equipped with an opening window and wood shaving bedding in the habitual horse training centre. Thermal and hygrometric records were carried out inside the box for the whole study using a data logger (Tinytag Ultra 2, TGU-4500, Gemini, London, UK). The temperature during the experimental period was 20.0 °C minimum and 24 °C maximum, and mean humidity was 55–60%. Animals were kept under a natural 12:12 light/dark (L/D) cycle (6:40 sunrise, 18:40 sunset) in Messina, Italy (Latitude: 38°11′43″; Longitude: 15°26′1″), at 150 m above sea level. The horses were fed alfalfa hay in equal quantities (4 kg) three times a day (at 7:00, 13:00, and 19:00 h), with water available ad libitum.

Total locomotor activity and heart rate were simultaneously recorded for a 24 h period, starting at 19:00 on day 1 and ending at 19:00 on day 2, during their weekly resting day. The total activity of horses, which includes different behaviours such as feeding, drinking, walking, grooming and all conscious and unconscious movements, was measured. To record activity, we equipped the animals with an Actiwatch-Mini^®^ (Cambridge Neurotechnology Ltd., Finstanton, UK), an actigraphy-based data logger that records a digitally integrated measure of motor activity, validated for locomotion scoring in large mammals [32]. This activity acquisition system is based on miniaturised accelerometer technologies, and Actiwatch utilises a piezo-electric accelerometer that is set up to record the integration of intensity, amount and duration of movement in all directions; it contains a single-axis accelerometer that records physical movement. The corresponding voltage produced is converted and stored as an activity count in the memory unit of the Actiwatch. The maximum sampling frequency is 32 Hz. Actigraphs were placed using a headstall accepted without obvious disturbance [22]. Activity was monitored with 5 min sampling intervals and expressed in arbitrary units. Also, the horses were equipped with an equine HR monitor (Polar Equine S-610I Polar^®^, Pacific Time, Milan, Italy) secured on an elastic band placed around the chest near the armpit. Two electrodes were placed on wet skin; the negative electrode was located in a region approximately facing the horse’s elbow, and the positive electrode was on the right side. The electrodes were connected to a transmitter (T51H) placed on the breast strap, which sent data to a watch-type data logger (Polar S-610) placed in the box drawer. Data were recorded every 5 s and later downloaded to a personal computer for analysis by Polar Equine 4.0 software.

### Statistical Analysis

All the results are expressed as the mean ± SD. Data were normally distributed (*p* < 0.05, Kolmogorov–Smirnov test). A trigonometric statistical model was applied to each time series to describe the periodic phenomenon analytically by characterising the main rhythmic parameters according to the single cosinor procedure [33], and four rhythmic parameters were determined, including mesor (mean level), amplitude (half the range of oscillation), acrophase (time of peak) and robustness (strength of rhythmicity). For each parameter, the mean level of each rhythm was computed as the arithmetic mean of all values in the data set (288 data points), and the amplitude of a rhythm was calculated as half the range of oscillation, which in its turn was computed as the difference between the peak and trough. Rhythm robustness (stationarity of a rhythm) was computed as the quotient of the variance associated with sinusoidal rhythmicity and the total variance of the time series [34]. To compare the locomotor activity recording with the heart rate recording, the mean of every 5 s data point for 5 min was computed for the heart rate data.

Pearson’s correlation test was performed to assess the correlation between the 24 h data point of total locomotor activity and heart rate for each horse. A linear regression model (y = a + bx) was applied to determine the degree of correlation between these parameters. *p* values < 0.05 were considered statistically significant. Data were analysed using the statistical software Prism v. 5.00 (GraphPad Software Ltd., Solana Beach, CA, USA, 2003).

## 3. Results

**Figure 1:**
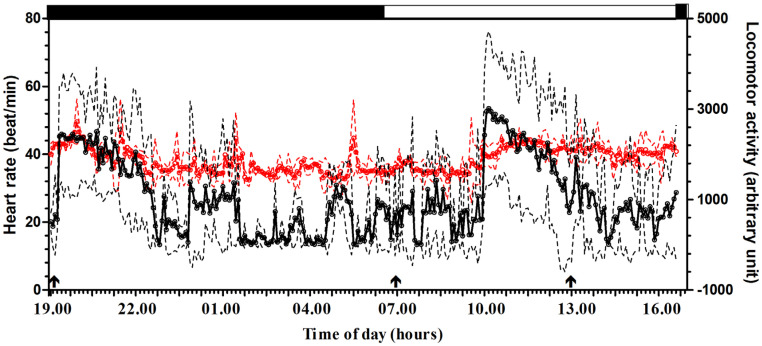
Trend of oscillation (mean ± SD) of total locomotor activity (black graph) and heart rate (red graph), expressed in their conventional units, recorded during the natural 12:12 light/dark cycle. Each point represents the mean recorded value (*n* = 10). The dotted lines represents the standard deviation of the mean. White and black bars indicate photophase and scotophase. The arrows indicate the time of feeding.

Figure 1 shows the mean ± standard deviation (SD) of locomotor activity (arbitrary unit) and heart rate (beats/min) recorded during the 24 h experimental period every 5 min.

**Figure 2:**
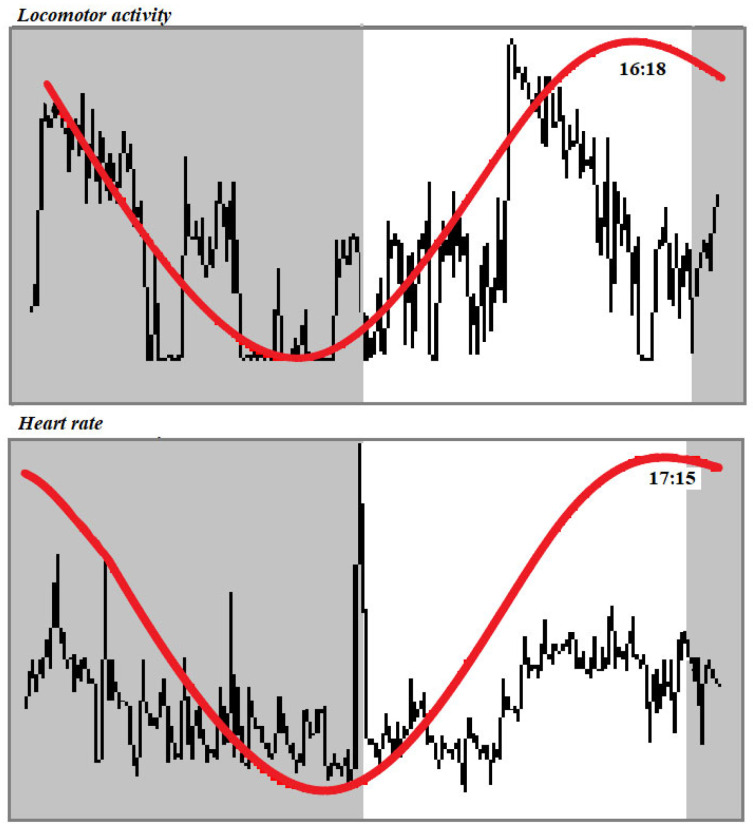
Acrophasogram of total locomotor activity and heart rate, expressed in their conventional units, recorded in a horse subjected to a natural 12:12 light/dark cycle. Grey bars indicate the dark phase of the natural photoperiod. The black line represents the value recorded at each data point during the 24 h. The red line represents the sinusoidal circadian rhythm.

**Figure 3:**
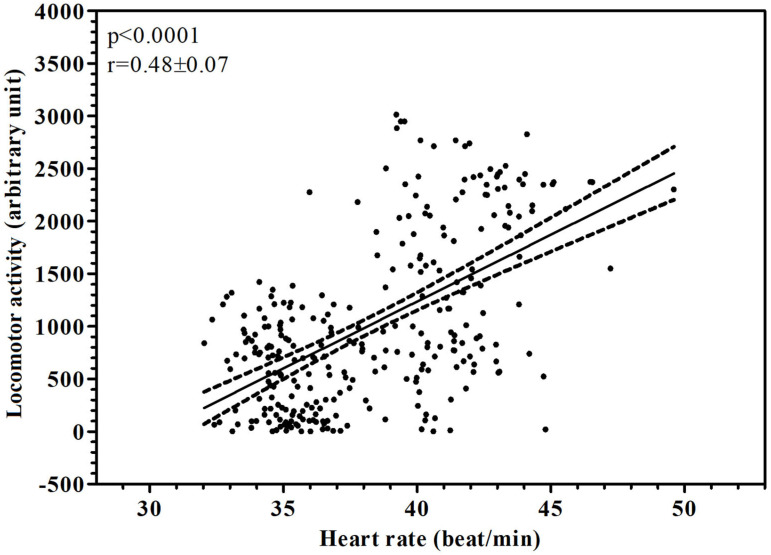
Correlation, and linear regression, together with the 95% limit of confidence of locomotor activity and heart rate, expressed in their conventional units, recorded in a horse subjected to a natural 12:12 light/dark cycle. Dash and solid represent linear regression and limit of confidence.

The application of the periodic model and statistical analysis of the cosinor enabled us to define the periodic parameters (mesor, amplitude, acrophase and robustness of rhythm). Both investigated parameters showed a daily rhythmicity with a diurnal acrophase (locomotor activity 17:05 ± 1:15; heart rate 16.79 ± 0.50). Figure 2 shows a representative acrophasogram (display of sets of acrophase) of the total locomotor activity and heart rate recorded in a horse during the experimental period. Mesor mean values were comprised between 531.31 and 1505.89 arbitrary units for locomotor activity and 38.02 and 38.64 beats/min for heart rate. Amplitude values were comprised between 255.41 and 831.36 arbitrary units and 2.68 and 4.62 beats/min. Robustness of the rhythm was between 10.50 and 25.40% and 36.60 and 37.50%, respectively, for the locomotor activity and heart rate (Table 1). A positive correlation was observed between the two investigated parameters in each horse, r = 0.48 ± 0.07, *p* < 0.0001 (Figure 3).

## 4. Discussion

Knowledge about behaviour and physiological patterns, including TLA and HR values, is of great importance for the optimum management of equines, as well as other domestic species. Modifications of the daily oscillation of these two parameters could be an index of discomfort due to housing and management conditions. The use of automated tools for recording the temporal trend of a specific parameter helps to establish reference values for these parameters and interpret their rhythmic pattern [35]. The continuous monitoring of TLA and HR has been previously performed in horses, but they have never been correlated in resting horses to verify their application in PLF. In particular, TLA is widely investigated in athletic horses, who are subjected to various natural environmental conditions and housing systems [18]. Our results are in accordance with previous findings showing that in stabled horses, TLA is mainly diurnal, with an acrophase in the middle of the photophase and low robustness values. Even though box stall confinement, limited and controlled exercise and concentrated feeding regimes in horses could be a distress for a social herd animal that is naturally free-roaming and pasture-grazing [36], it has been supposed by the analysis of circadian rhythm parameters of TLAs recorded in horses with different sport attitudes for ten consecutive days that the routine procedures that are performed in stall management act as an entrainment (synchronisation) signal for daily rhythms [37]. The continuous monitoring of HR every 5 s gives a more precise fluctuation of this parameter during the 24 h with respect to recording by technician auscultation. It is an extremely variable parameter affected by the temporary response of horses to sudden environmental incidents [38]. A study about horses’ chronocardiology revealed a rhythmicity of heart rate at different frequencies, featuring circadian and circasemidian rhythms with nocturnal acrophase [39]. Our results showed a diurnal rhythm of heart rate with an acrophase in the middle of the photophase and middle robustness values. Several studies have been conducted to correlate equine TLA with other physiological parameters, such as rectal temperature and cortisol; nevertheless, current knowledge about the correlation with heart rate is limited. Deviations in movement patterns or abnormal HR levels can signal stress, illness or discomfort, thereby helping to identify and address welfare issues proactively. TLA reached its acrophase at 16:18, and it was followed about an hour later by an HR acrophase at 17:15. Different rhythms peak at different times of the day, so that one can find at least one rhythm in the body peaking at any given time of the day. This raises the issue of internal order in organisms—that is, how the various processes in the body relate to each other. This relationship between the two parameters was confirmed by the application of Pearson’s correlation test, showing a positive correlation between the two parameters. A positive correlation between TLA and HR has been previously found in freely moving rats [40] and in river shrimp [41]. The reason of this correlation is not clear yet. Heart rate varies with changes in activity, posture and other external stimuli. The simultaneous study of many physiological variables is a necessary step to understand the temporal relationships of physiological processes. TLA and HR showed different robustness values. This finding is not surprising, as TLA in horses showed low robustness value in various experimental conditions [36]. For the issue of causality, a rhythm with low robustness cannot be the cause of a rhythm with high robustness. Thus, the rhythms of HR must not be caused by the rhythm of TLA. In the analysis of this correlation, one could take into consideration that horses of different sporting attitudes had a different response to noxae, observed not only among the subjects but also within subjects, recordable by the application of an accelerometer recording the TLA [22]. Heart rate recording is a good indicator of parasympathetic activity [38]. The parasympathetic nervous system is proposed as the modulator of stress vulnerability and reactivity. Therefore, the quantification of heart rate oscillations is described as a method for assessing the stress response in mammals [38]. Continuous monitoring of TLA and HR is important for the assessment of horse well-being, which is essential for conservation efforts. By providing insights into energy expenditure and physiological responses, these metrics help in the understanding of how animals adapt to their environments, directly impacting their management and traditional practise. The variability in responses could be influenced by factors like environmental conditions, psychological stress and individual temperament or overall age and health [31].

## 5. Conclusions

Our study was conducted for a limited amount of time and in only experimental conditions; our results can be considered a starting point for further investigation regarding the persistence of this correlation in other experimental conditions and the reason for this correlation. The use of a device for continuous recording during the 24 h makes this assessment more precise. The application of new technologies for the simultaneous recording of physiological indexes of animals’ welfare can also be a useful instrument in equine management to verify discomfort situations due to housing and management conditions.

## Figures and Tables

**Table 1:** Mean ± standard deviation (*n* = 10) of rhythmic parameters expressed in their conventional units.

	Mesor	Amplitude	Acrophase (h·min)	Robustness (%)
Total locomotor activity (arbitrary unit)	1018.60 ± 689.13	543.38 ± 407.25	17.05 ± 1.15	17.95 ± 10.53
Heart rate (beats/min)	38.33 ± 0.43	3.65 ± 1.37	16.40 ± 0.30	37.05 ± 0.63

## Data Availability

The raw data supporting the conclusions of this article will be made available by the authors on request.

## Abbreviations

The following abbreviations are used in this manuscript:

TLA	Total locomotor activity
HR	Heart rate
PLF	Precision livestock farming
